# Editorial: Low-fat and low-salt cheeses: Technological strategies to improve the nutritional profile and sensory characteristics

**DOI:** 10.3389/fnut.2023.1155604

**Published:** 2023-03-30

**Authors:** Carina Bergamini, Erica Hynes, Antonio-José Trujillo, María Cristina Perotti

**Affiliations:** ^1^Instituto de Lactología Industrial, Consejo Nacional de Investigaciones Científicas y Técnicas (CONICET), Universidad Nacional del Litoral (UNL), Santa Fe, Argentina; ^2^Centre d'Innovació, Recerca i Transferència en Tecnologia dels Aliments (CIRTTA), CERTA-TECNIO, Department of Animal and Food Science, Faculty of Veterinary Sciences, Universitat Autònoma de Barcelona (UAB), Bellaterra, Spain

**Keywords:** low-fat cheeses, low-salt cheeses, technological strategies, sensory characteristics, nutritional profile, cheese ripening

Dairy foods, especially fermented products, are positively valued by consumers both from sensory and nutritional viewpoints, and they provide highly bioavailable calcium and proteins. Cheeses share those positive aspects and also shows two drawbacks: high saturated fat and high sodium. High intakes of these nutrients are related to chronic and non-transmissible pathologies such as obesity, hypertension, and cardiovascular diseases. Over the last decades, awareness has risen worldwide about the relation between diet and health, and both consumers and industry mind food composition (1).

Different strategies have been proposed to reformulate cheeses for fat and salt reduction, improving their nutritional profile. However, decreasing these components leads to diminished sensory characteristics, mainly texture and flavor, and could also affect microbial safety. In this sense, salt plays important roles in the cheese properties through multiple functions as it imparts taste and aroma, regulates the water activity, the microbial growth (starter and non-starter bacteria and adventitious microorganisms), the enzymatic activity and biochemical changes during ripening, and influences the casein hydration and its susceptibility to proteolysis, among others. Fat, on the other hand, has a main influence on the texture, aroma, and taste of cheeses. Therefore, the cheese making of good quality and highly acceptable low-fat and/or low-salt cheeses represents a great challenge for the dairy industry (2, 3).

This Research Topic is aimed at collecting studies about the application of different strategies to obtain healthier cheeses, with lower fat and salt contents, and with good global quality and consumer acceptability. In this special e-collection, there are four articles covering some of the aspects related to salt reduction in cheeses, which are described later, while no articles were received about the strategies for the reduction of fat in cheese and the impact on quality. The main strategies reported about the last topic have been focused on increasing moisture retention through different changes in the cheese making, including the addition of different ingredients, such as protein enrichment of the milk, especially with microparticulated whey proteins (4, 5) or with the use of ultrafiltrated milk. In addition, diverse polysaccharides were also used as fat mimetics such as sodium carboxymethyl cellulose and waxy rice starch (6) and inulin (7), among others. Other strategies involved the modification of the cheese proteins by the treatment with transglutaminase (6) or the modification of the fat structure by homogenization (8). Finally, adjunct cultures were used to improve the flavor of fat-reduced cheeses (4).

Regarding the strategies to obtain sodium-reduced cheeses, the mini-review entitled “*The reduction of salt in different cheese categories: recent advances and future challenges*,” by Tidona et al., summarizes the salt reduction strategies for soft, semi-hard, hard, and mold-ripened cheeses. This article highlights that any modification in the salting procedure needs to be investigated for each type of cheese to ensure a regular ripening process, sensory acceptance, and the safety of the product. The decrease in NaCl and its partial replacement by KCl have been the most successful and best-characterized strategies for several types of cheeses. The study of Juan et al., “*The effect of salt reduction and partial substitution of NaCl by KCl on physicochemical, microbiological, sensorial and consumers' acceptability of semi-hard and hard lactose free cow's milk cheeses*,” showed that the reduction in salt (>25%) by different brine conditions (half the salting time and partial substitution of NaCl by KCl in the brining composition) did not affect the physicochemical characteristics (pH and dry matter), microbial growth, proteolysis, texture, and color of cheeses. Similarly, Gagnaire et al., in the article entitled “*Little impact of NaCl reduction in Swiss-type cheese*,” demonstrated that the reduction in the NaCl by 30% did not change the Swiss-type cheese characteristics.

Other approaches are based on the modification of the salinity perception through changes in the textures and structure of the food matrix and by the incorporation of different components, which produce sensory cross-interactions (9, 10). In this sense, the use of polysaccharides could influence salt perception through cheese texture modification. In addition, the fat content showed an inverse relation with the salinity, which could be associated with changes in the cheese texture or also with a barrier effect of fat toward the salt release in the mouth (10, 11). On the other hand, the incorporation of flavor enhancers (monosodium glutamate, ribonucleotides, hydrolyzed vegetable protein, and yeast extract), bitter blocked agents (glycin, lysin, taurine, and adenosine-5'monofosfate), and aroma compounds (such as aromatic species) has shown a potential for the obtention of good quality low-salt foods. The incorporation of cultures able to produce compounds with these properties is another suitable option. In the article of Juan et al., the addition of yeast extract as a flavor enhancer to semi-hard and hard cheeses led not only to a higher salt but also to a higher bitter note and aftertaste, which negatively impacted consumer acceptability. It is needed to find suitable levels of these ingredients to obtain favorable results for each case and to study whether the microbiota could metabolize them and produce other compounds with a positive or negative impact on the cheese flavor. In the article “*Reducing salt content in real cheeses while increasing salty taste and fat perception using aroma*,” Syarifuddin et al. verified an enhancement of the saltiness when sardine aroma (salt-associated) was added to cheeses. The same effect, but to a lesser extent, was found for butter aroma (fat-associated). In addition, a blend of these two aroma compounds led to an enhancement of fat perception. The mechanisms of sensorial interactions are complex and more studies are needed to understand them. The reaction of consumers to including these ingredients and displaying them on the product label should also be explored (Tidona et al.).

The choice of starter cultures and coagulants, and the adaptation of the cheese making technology offer opportunities to counteract, at least partially, the several drawbacks detected by reducing salt content in cheese but depending on cheese variety (Tidona et al.). In this way, Gagnaire et al. showed that some properties of Swiss-type cheeses were more affected by highly proteolytic *Lactobacillus helveticus* strains than by the decrease of 30% of salt. The reduction in salt could also affect the microbial lysis and the release of enzymes that act during ripening; this highlights the importance of a suitable selection of starter in reduced-salt cheeses (Gagnaire et al.). Regarding the microbial safety of reduced-salt cheeses, protective cultures, which produce inhibitory compounds or exert a competitive inhibition, or the addition of natural preservative compounds could replace the inhibitory effect of the salt on undesirable microorganisms (Tidona et al.).

To summarize, the reduction in salt in cheese must be carried out with care because salt has multiple functions; the effectiveness of the individual or combined strategies with this objective should be assayed in each type of cheese because the impact could be different. Many aspects remain to be explored, such as the application of nanotechnology, which is one of the most innovative and least explored strategies. Research on the market and consumer trends should also be considered when new products are developed. In addition, a better understanding of the sensorial interactions would allow the design of effective strategies to maintain cheese acceptability while reducing salt and fat content.

## Author contributions

All authors listed have made a substantial, direct, and intellectual contribution to the work and approved it for publication.
